# Core-multishell globular oxidation in a new TiAlNbCr alloy at high temperatures

**DOI:** 10.1038/s41598-017-03690-0

**Published:** 2017-06-14

**Authors:** S. Q. Tang, S. J. Qu, A. H. Feng, C. Feng, J. Shen, D. L. Chen

**Affiliations:** 1 School of Materials Science and Engineering, Tongji University, Shanghai, 201804 P.R. China; 2 Department of Mechanical and Industrial Engineering, Ryerson University, Toronto, Ontario M5B 2K3 Canada

## Abstract

Oxidation resistance is one of key properties of titanium aluminide (TiAl) based alloys for high-temperature applications such as in advanced aero-engines and gas turbines. A new TiAlNbCr alloy with micro-addition of yttrium has been developed, but its oxidation behavior is unknown. To provide some fundamental insights, high-temperature oxidation characteristics of this alloy are examined via scanning electron microscopy, transmission electron microscopy, electron probe microanalysis, and X-ray diffraction. We show that distinctive core-multishell globular oxidation and “daisy” flower-like oxidation occur exclusively around Y_2_O_3_ particles. Globular oxides exhibit multi-layered Y_2_O_3_/TiO_2_/Al_2_O_3_-rich/TiO_2_-rich shell structures from the inside to outside. Flower-like inner oxides consist of core Y_2_O_3_ particles surrounded by divergent Al_2_O_3_ and oxygen-rich α_2_-Ti_3_Al in the near-scale substrate. As the scale-substrate interface moves inward, the inner oxide structures suffer deeper oxidation and transform into the globular oxide structures. Our results demonstrate that the unique oxidation characteristics and the understanding of formation mechanisms pave the way for the exploration and development of advanced oxidation-resistant TiAl-based materials.

## Introduction

Titanium aluminide (TiAl) based alloys are considered to be a new class of promising advanced high-temperature structural materials in the aerospace, gas turbine and automotive industries, because of their lightweighting with a low density (3.9–4.2 g/cm^3^), high specific yield strength and stiffness, and superior creep resistance at elevated temperatures^1–6^. This is highly inspired by the recent successful application of a TiAl-based alloy in General Electric’s high-thrust GEnex jet engines for powering Boeing 747-8 and 787 Dreamliner, to substitute Ni-based superalloys in the temperature range of 650–750 °C with the benefit of a weight reduction of ~50%^1–3^. However, their wide commercial applications are still limited due to low room-temperature ductility^7, 8^, lack of a cost-effective processing method, and unsatisfactory oxidation resistance at temperatures above 750 °C^9, 10^. Unlike Ni-Al alloys, no protective Al_2_O_3_ layer could occur on the titanium aluminide alloys because both titanium and aluminum form oxides of similar stability^11^. Therefore, many measures were taken to improve the oxidation resistance of titanium aluminide alloys such as surface treatment^12, 13^ and coating technologies^14–16^. In particular, both mechanical properties and oxidation resistance at high temperatures can be simultaneously improved via adding moderate ternary or quartic elements such as niobium^17^, tungsten, silicon, molybdenum^18^, chromium^19^ and yttrium^20^ in binary titanium aluminide alloys^21, 22^. Yttrium exhibits a strong grain refinement effect, thus improving the tensile strength of TiAl-based alloys^23, 24^. The addition of yttrium also significantly improves oxidation resistance due to its strong affinity to oxygen^20, 23, 25, 26^. Oxide pegs protruding into the substrate^26^ and convex-shaped nail-like oxides^25^ were observed at the scale-substrate interface, which play an important role in anchoring the oxide scale and improving anti-spalling ability of the scale on the surface of titanium aluminide alloys with an addition of yttrium. However, it is unclear how and in which form the oxidation occurs at high temperatures. In the present study, a new type of core-multishell globular oxide structure consisting of different oxide layers formed in a newly-developed Ti-44Al-4Nb-1.5Cr-0.5Mo-0.1B-0.1Y alloy induced by the presence of yttrium oxide (Y_2_O_3_) particles during oxidation at a high temperature of 900 °C is observed and discussed. Special attention is paid to the formation and growth mechanism of this unique type of globular oxides to give underlying insights about the effect of yttrium on the oxidation process of titanium aluminide alloys.

## Results

### Microstructures of TiAlNbCr alloy

**Figure 1:**
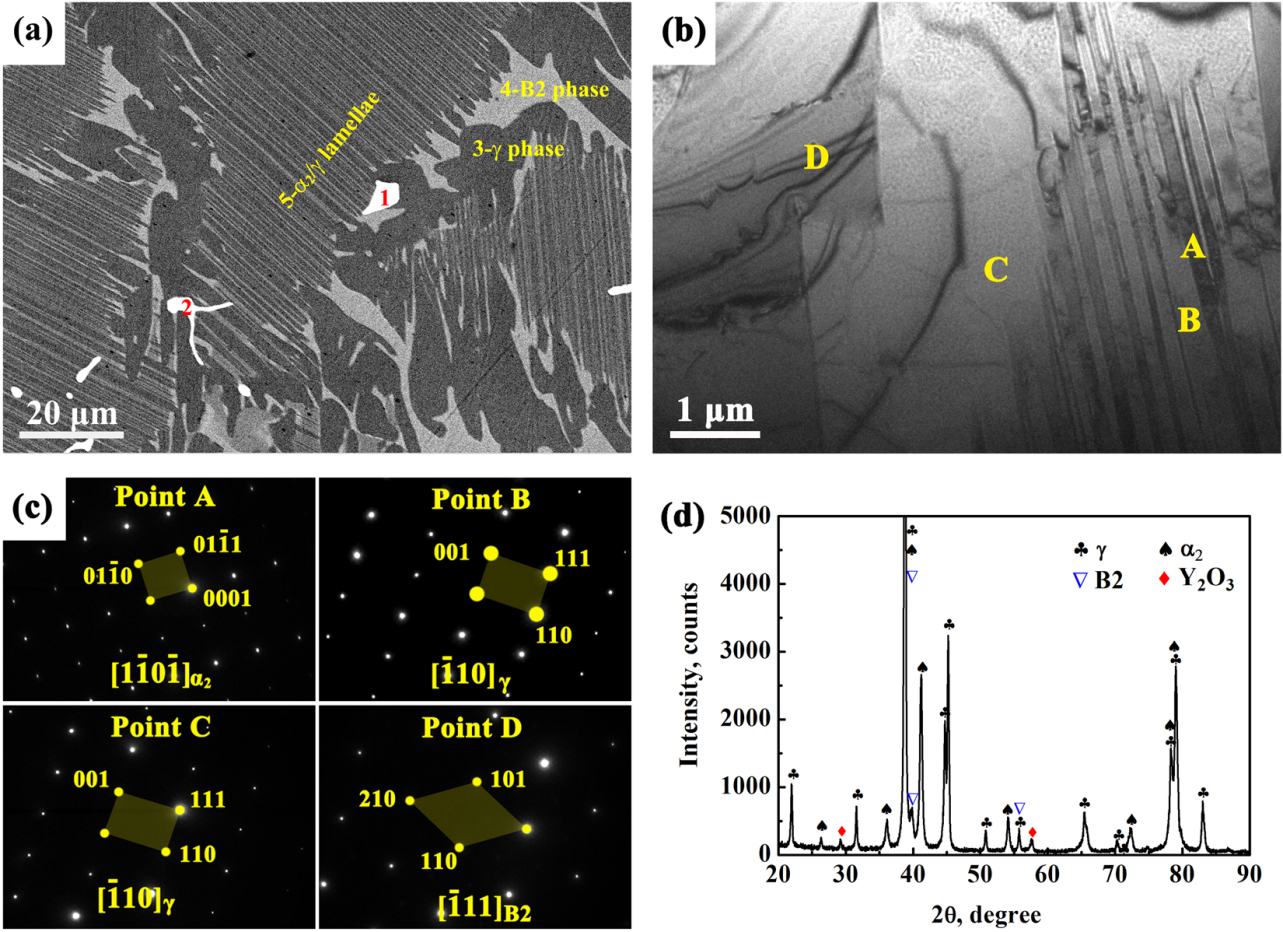
Microstructures of as-cast TiAlNbCr alloy. (**a**) SEM back-scattered electron micrograph showing gray α_2_-Ti_3_Al and dark γ-TiAl lamellae, white B2-phase, and bright white Y_2_O_3_ particles, (**b**) TEM bright field image along with (**c**) the corresponding selected area diffraction (SAD) patterns of points A-D, where A represents α_2_-Ti_3_Al lamella, B corresponds to γ-TiAl lamella, C indicates γ-TiAl, and D stands for B2, and (**d**) XRD pattern further confirming the presence of α_2_-Ti_3_Al, γ-TiAl, B2-phase and Y_2_O_3_ particles.

Back-scattered electron (BSE) SEM micrograph, TEM bright field image along with the relevant selected area diffraction (SAD) patterns, and XRD pattern of as-cast TiAlNbCr alloy are shown in Fig. 1(a) through (d). The elemental compositions of points marked by “1” to “5” in Fig. 1(a) via electron probe microanalysis (EPMA) are summarized in Table 1. These analyses revealed that the microstructure consisted mainly of white B2-phase, dark γ-TiAl phase at colony (or grain) boundaries, which has an ordered L1_0_ structure, gray α_2_-Ti_3_Al, which has an ordered hexagonal D0_19_ structure, and α_2_/γ lamellar colonies as identified by the SAD and XRD patterns in Fig. 1. This is also corroborated via EPMA point chemical microanalyses, where points “3”, “4” and “5” corresponded to γ-TiAl, ordered titanium with B2 structure and α_2_/γ lamellar colony, respectively. The disordered β phase existing at high temperatures, which exhibits multiple slip systems due to its body-centered cubic crystal structure, will transform into the brittle B2-ordered phase in Ti-Al alloys at room temperature^5, 22^. Therefore, the volume fraction of B2 phase should be carefully controlled. Points “1” and “2” and other bright white particles imbedded in α_2_/γ lamellar colonies were identified to be Y_2_O_3_ particles. However, YAl_2_ compounds reported in other TiAl-based alloys with yttrium addition^26^ were not observed in the present TiAlNbCr alloy.

**Table 1:** Elemental composition (at.%) determined via EPMA at points 1–5 in Fig. 1(a).

Number	Ti	Al	Nb	Cr	Mo	B	Y	O	N
1	4.94	3.27	0.29	0.12	0.03	0	38.19	53.16	0
2	4.95	3.63	0.31	0.17	0.01	0	38.10	52.84	0
3	49.02	45.62	4.32	0.92	0.12	0	0	—	—
4	58.26	31.92	5.14	3.65	1.04	0	0	—	—
5	52.91	41.82	3.86	1.23	0.19	0	0	—	—

### Isothermal oxidation kinetics

**Figure 2:**
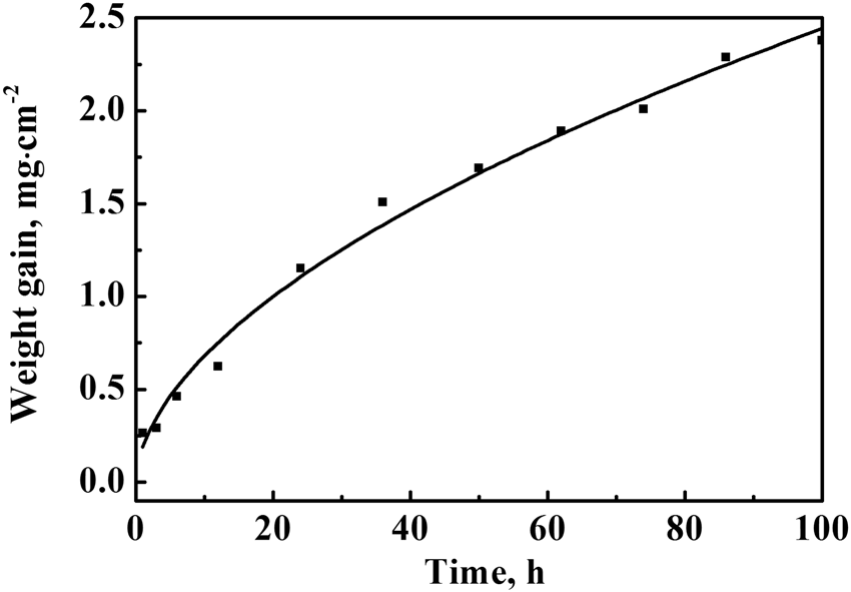
Oxidation kinetics of TiAlNbCr alloy at 900 °C up to 100 h. Weight gain per unit area as a function of time.

Figure 2 shows a curve of isothermal oxidation kinetics of TiAlNbCr alloy at 900 °C. The obtained weight gain of this alloy after 100 h at 900 °C was about 2.3777 mg/cm^2^. To identify which law of oxidation kinetics is followed, the obtained experimental data could be fitted using the following equation,

1 $${\rm{\Delta }}{M}^{n}={k}_{n}t,$$

where *∆M* represents weight gain per unit area (mg/cm^2^), *n* is an oxidation exponent (*n* = 1, linear relationship; *n* = 2, parabolic relationship), *k*_*n*_ is a rate constant (mg^n^/cm^2n^ h) and *t* is oxidation time (h). The obtained oxidation exponent was close to 2, suggesting that the oxidation kinetics of TiAlNbCr alloy at 900 °C obeyed a parabolic relationship.

### Core-multishell globular oxidation

**Figure 3:**
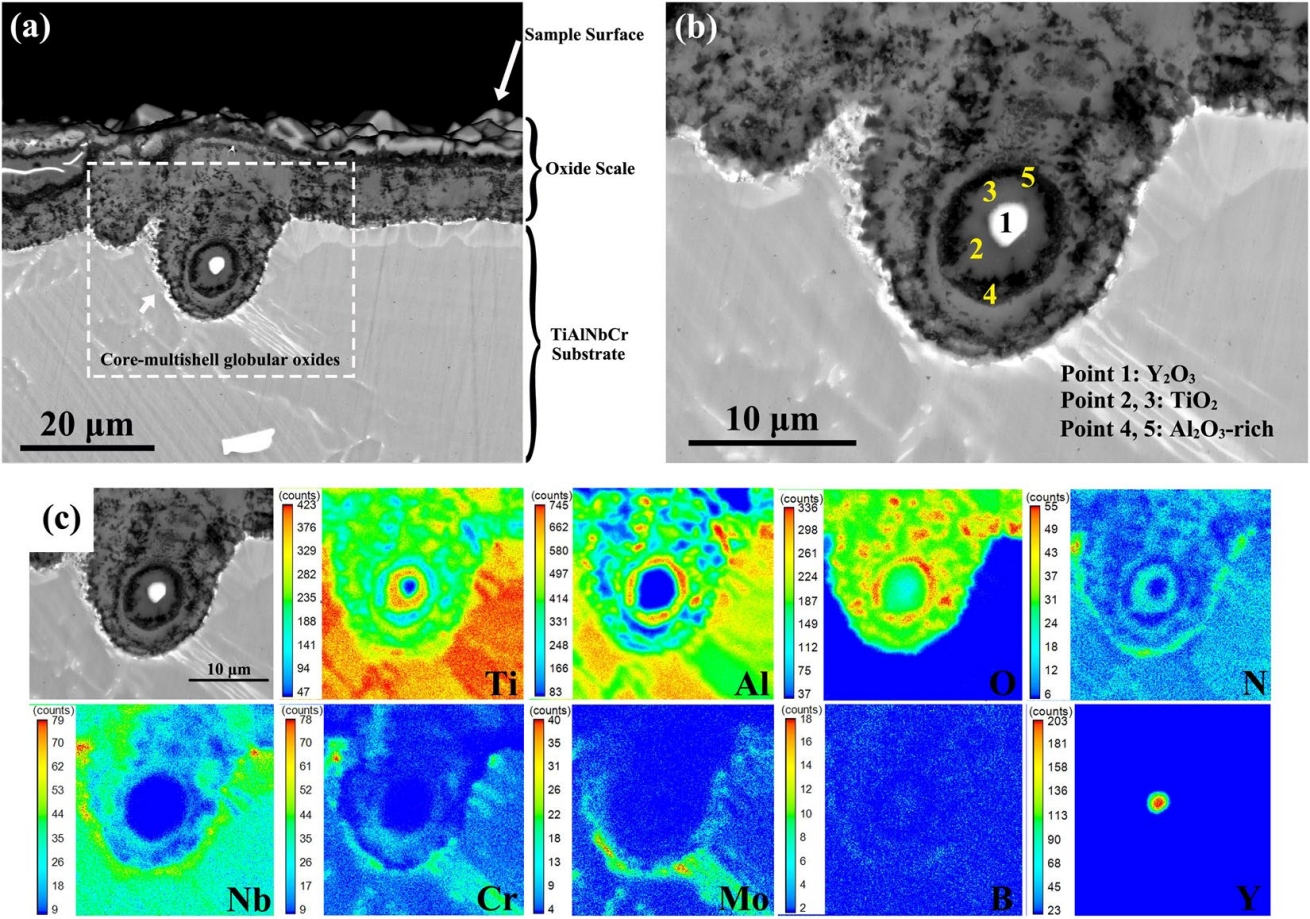
A typical core-multishell globular oxide of TiAlNbCr alloy after oxidation at 900 °C for 100 h. (**a**) and (**b**) Morphologies of oxides with a globular structure, which consist of a core Y_2_O_3_ particle and multi-layered TiO_2_/Al_2_O_3_-rich/TiO_2_-rich shells from the inside to outside, and (**c**) elemental distributions via EPMA mapping for the globular oxide, where the detailed chemical microanalysis results are shown in Table 2.

**Figure 4:**
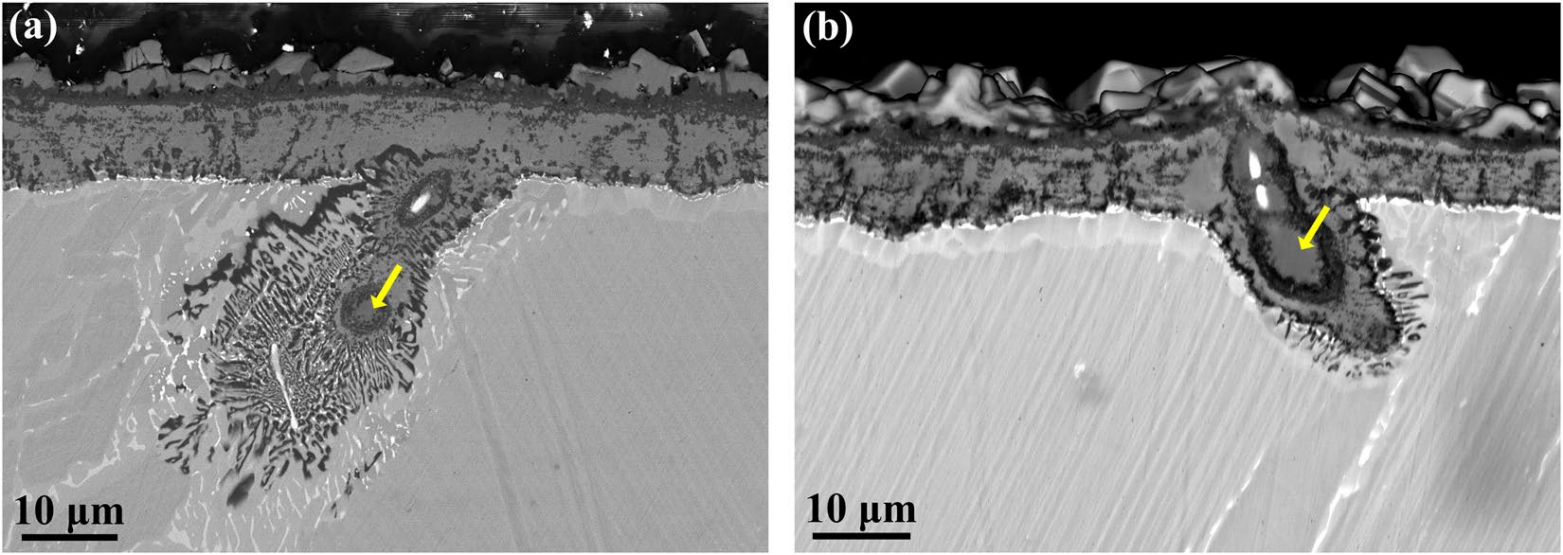
Typical micrographs showing transient core-shell globular oxides in TiAlNbCr alloy after oxidation at 900 °C for 100 h. (**a**) and (**b**) Morphologies of two oxides with a transient or incomplete core-shell globular structure at an intermediate distance to the scale-substrate interface.

Figure 3(a) and (b) shows the cross-sectional morphology of a typical oxide with globular structures after oxidation at 900 °C for 100 h. The globular oxides were normally located inside or underneath the oxide scale and they were also observed in all other specimens after oxidation at 900 °C for 12, 24 and 50 h. The bright white core of the globular oxide marked by “1” in Fig. 3(b) was identified to be Y_2_O_3_ according to EPMA point chemical microanalysis results shown in Table 2 and EPMA elemental distribution maps in Fig. 3(c). The adjacent gray loop marked by “2” and “3” and outer dark loop marked by “4” and “5” in Fig. 3(b) were confirmed as TiO_2_ and Al_2_O_3_-rich shells, respectively. The outermost layer of TiO_2_-rich shell in this globular oxide contacted with the TiO_2_+Al_2_O_3_ mixture layer of the oxide scale. Therefore, the globular oxide structure consisted of a core Y_2_O_3_ particle and multi-layered TiO_2_/Al_2_O_3_-rich/TiO_2_-rich shells from the inside to outside. This kind of oxides protruding into the substrate could improve the adhesion of scale^25, 26^. It should be noted that transient or incomplete core-shell globular oxidation could also be observed, as shown in Fig. 4. This was mainly dependent on the oxidation time or the location of Y_2_O_3_ particles (i.e., the distance of Y_2_O_3_ particles to the scale-substrate interface). Additionally, while some Y_2_O_3_ particles were embedded or buried beneath the polished surface, as indicated by arrows in Fig. 4(a) and (b), they still induced the core-shell like globular oxidation. Such embedded Y_2_O_3_ particles were expected to be shallowly positioned just under the surface skin.

**Table 2:** Elemental composition (at.%) determined via EPMA at 1–5 points in Fig. 3(b).

Number	Ti	Al	Nb	Cr	Mo	B	Y	O	N	Remarks
1	7.89	0.21	0.034	0.05	0.03	0	36.42	55.37	0	Y_2_O_3_ (bright white)
2	31.56	1.25	0.02	0.17	0.06	0	0.18	62.02	4.75	TiO_2_ (gray)
3	27.11	7.74	0.06	0.20	0.06	0	0.35	60.27	4.22	
4	12.31	22.31	0.23	0.59	0.08	0	0.35	62.79	1.35	Al_2_O_3_-rich (dark)
5	12.07	23.55	0.51	0.35	0.04	0	0.22	62.17	1.10

### “Daisy” flower-like inner oxidation

**Figure 5:**
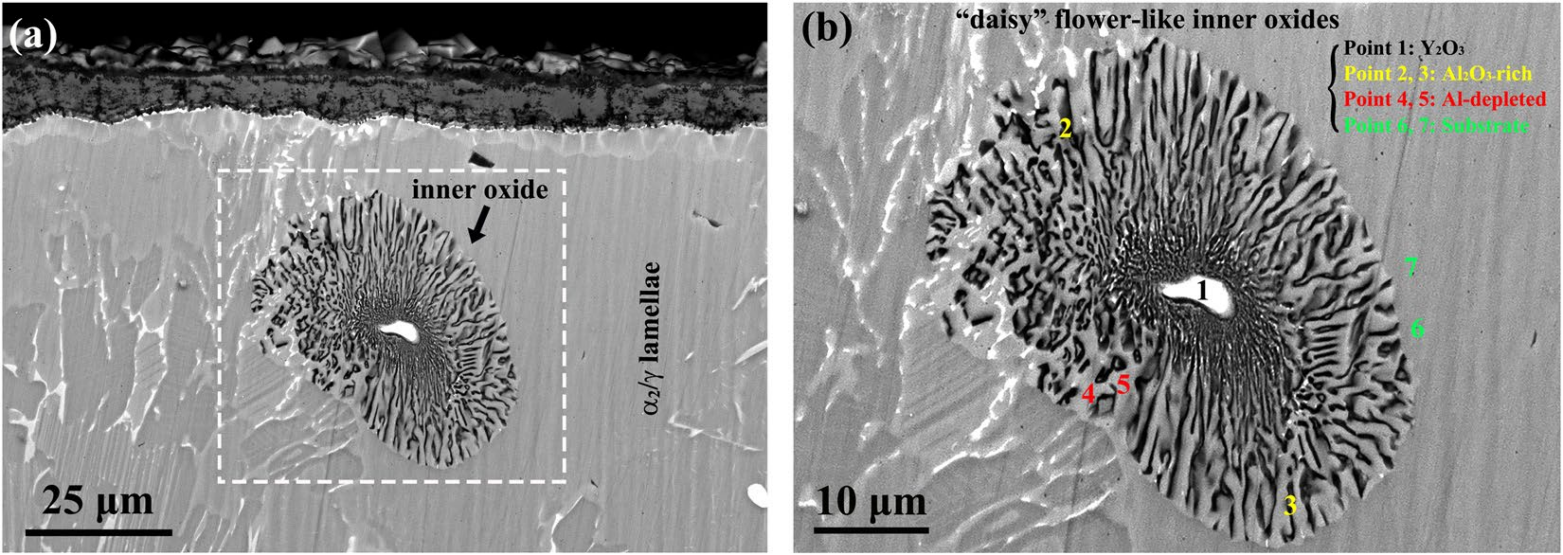
“Daisy” flower-like morphologies of an inner oxide after oxidation at 900 °C for 50 h. (**a**) A low-magnification image showing the location of inner oxide with respect to the scale-substrate interface, and (**b**) a magnified image of Fig. 3(a) showing the oxide details consisting of a core Y_2_O_3_ particle surrounded by divergent Al_2_O_3_ and oxygen-rich ${\alpha }$_2_-Ti_3_Al, where the EPMA microanalysis locations are marked by “1”–“7” with the obtained results summarized in Table 3.

It is also of special interest to observe a unique type of “daisy” flower-like inner oxides present in the alloy substrate close to the oxide scale (but without linking to the scale-substrate interface), as shown in Fig. 5(a) and (b). This was unlike the situation of inner oxides in other alloys, such as intergranular internal oxides and internal-oxide bands^27^. In the present study, all the inner oxide structures exclusively centered on a bright white particle. EPMA point chemical microanalysis results of points marked by “1”–“7” in Fig. 5(b) were summarized in Table 3 and the bright white core marked by “1” was again determined to be a Y_2_O_3_ particle. At points “2” and “3”, the concentration of aluminum was higher than that of titanium, which was in contrast to points “6” and “7” in the lamellar colony of substrate. This means that aluminum with a higher chemical activity at high temperatures was preferentially oxidized to form the divergent inner Al_2_O_3_ oxides around Y_2_O_3_ particles in the substrate. Points “4” and “5” close to the inner oxides were speculated as α_2_-Ti_3_Al rich in oxygen. This was likely due to the fact that (1) the formation of inner Al_2_O_3_ consumed aluminum which led to an increase of the relative concentration of titanium; (2) the saturation concentration of oxygen in the α_2_-Ti_3_Al and γ-TiAl alloys was calculated to be 16% and 2%, respectively^28^, and the oxygen concentration at points “4” and “5” were about 23.6% and 18.9% which were closer to 16%. However, the inner oxides embedded in the substrate may be detrimental to the mechanical properties^29, 30^.

**Table 3:** Elemental composition (at.%) determined via EPMA at points 1–7 in Fig. 5(b).

Number	Ti	Al	Nb	Cr	Mo	B	Y	O	N	Remarks
1	3.99	1.28	0.24	0.04	0	0	36.91	57.54	0	Y_2_O_3_ (bright white)
2	16.46	31.68	1.26	1.00	0.06	0	0	46.72	2.82	Al_2_O_3_-rich (dark)
3	21.58	24.06	1.55	0.37	0.08	0	0.01	50.59	1.77	
4	40.98	24.66	2.36	1.06	0.12	0	0	23.63	7.19	Al-depleted (gray)
5	44.64	23.63	3.44	0.85	0.22	0	0	18.92	8.30	
6	43.41	32.48	3.08	0.88	0.17	0	0	9.17	10.80	Substrate
7	41.93	32.91	3.10	0.96	0.15	0	0	8.91	12.05	

## Discussion

Yttrium with a low concentration of 0.1% was added in our TiAlNbCr alloy aiming to remove the oxygen which was present in the alloy in the process of smelting. This goal was successfully achieved due to the reaction of yttrium with oxygen to form Y_2_O_3_ particles, as shown in Fig. 1(a). After high-temperature oxidation at 900 °C, Y_2_O_3_ particles were still stable and remained unchanged. However, they were observed to induce high-temperature oxidation and occupy the cores of both globular oxide structures and “daisy” flower-like inner oxide structures. Globular oxides were positioned inside or underneath the oxide scale on the surface, which were directly linked to the scale (Fig. 3), while the characteristic flower-like inner oxidation occurred within the substrate in the vicinity of scale-substrate interface (Fig. 5). This suggests that the area surrounding Y_2_O_3_ particles was susceptible to oxidation at a high temperature of 900 °C. As the oxidation continued, the scale-substrate interface moved inwards and flower-like inner oxide structures were increasingly oxidized and eventually become the globular oxide structures (Fig. 4). It was as if the “daisy” flower-like inner oxide structures were the precursor of the globular oxide structures and the Y_2_O_3_ particles were the seeds of oxidation at high temperatures.

**Figure 6:**
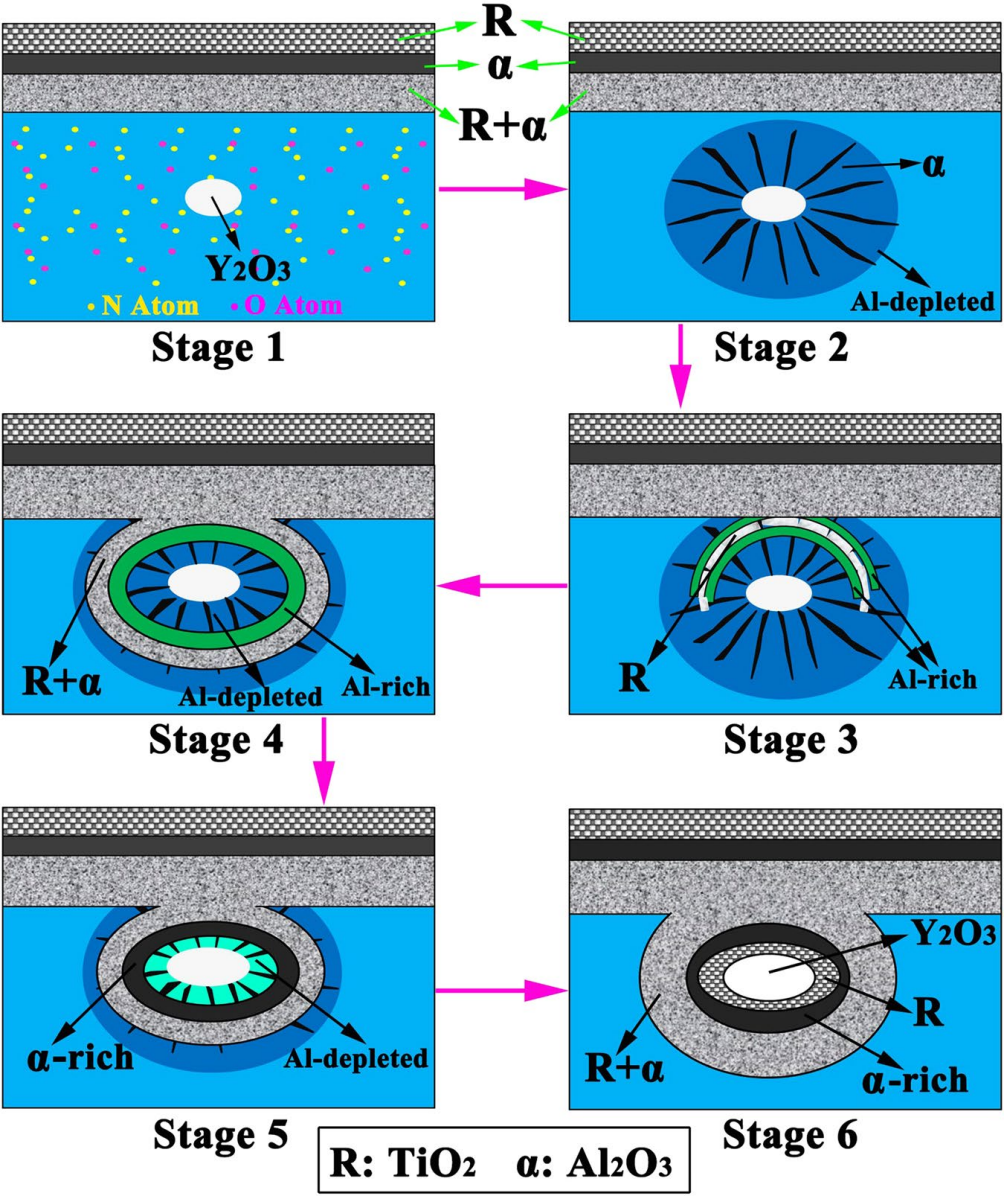
Formation and growth process of a distinctive core-multishell globular oxide in the TiAlNbCr alloy during high-temperature oxidation. Stage 1, inward diffusion of oxygen and nitrogen; Stage 2, aluminum reacted with the penetrated oxygen preferentially near the interface between Y_2_O_3_ particle and substrate to form a flower-like inner oxide; Stage 3, formation of a partial TiO_2_-rich shell mingled with a small amout of Al_2_O_3_; Stage 4, formation of a full TiO_2_-rich shell mingled with Al_2_O_3_, along with an inner aluminum-rich shell; Stage 5, formation of an Al_2_O_3_ shell; and Stage 6, formation of the innermost TiO_2_ shell.

Figure 6 illustrates the formation and growth mechanism of the core-multishell globular oxide structures in the TiAlNbCr alloy during high-temperature oxidation. First, a thin oxide scale formed on the surface in conjunction with the inward diffusion of oxygen and nitrogen as seen in “Stage 1”. Then aluminum, which was more active than titanium at a high temperature of 900 °C as seen from their standard free energy of oxidation as a function of temperature^31^, reacted with the penetrated oxygen preferentially near the interface between Y_2_O_3_ particles and substrate to form flower-like inner oxides as shown in Fig. 5. Al_2_O_3_ firstly formed at the Y_2_O_3_-substrate interface and the reasons could be considered as follows:

- The Y_2_O_3_-substrate interfacial energy could be lowered when the nucleation of Al_2_O_3_ occurred there.
- The local tensile stresses existent in the nearby substrate arising from the nucleation and growth of Y_2_O_3_ could drive the formation of inner oxides^32^.
- The tensile stresses present at the Y_2_O_3_-substrate interface could result in the formation of microvoids or other defects at the interface which provided a growing space of Al_2_O_3_ or fast diffusion channel of oxygen^27, 32^.

With the consumption of aluminum, α_2_-Ti_3_Al rich in oxygen formed in the Al-depleted areas beside divergent inner Al_2_O_3_ products as shown in “Stage 2”. In “Stage 3”, the scale-substrate interface continued to move inward and contacted with the flower-like inner oxide. Then TiO_2_ formed preferentially in α_2_-Ti_3_Al(O)^33^ perpendicular to divergent Al_2_O_3_ along with two aluminum-rich layers. The previously formed Al_2_O_3_ was thus broken up and dissolved into TiO_2_ lattice, because of the volume expansion caused by the formation of TiO_2_^34^. The so-called fast-grown TiO_2_^35^, rapidly moved around Y_2_O_3_ particle to form a full TiO_2_-rich shell mingled with a small amount of Al_2_O_3_, along with an inner aluminum-rich shell as shown in “Stage 4”. In the subsequent oxidation in “Stage 5”, Al_2_O_3_ preferentially grew in the inner aluminum-rich shell because of the higher relative activity of aluminum, leading to an Al_2_O_3_-rich shell as seen at points “4” and “5” in Fig. 3(b), and the formation of an aluminum-depleted shell directly adjoining to the central Y_2_O_3_ particle. Finally, in “Stage 6” this innermost aluminum-depleted shell was replaced by TiO_2_ as oxygen diffused inward via a certain extent of break-up and dissolution of the previously-formed neighboring Al_2_O_3_ oxides in the form^36^,

2 $${{\rm{Al}}}_{2}{{\rm{O}}}_{3}=2{{\rm{Al}}}^{3+}+\tfrac{3}{2}{{\rm{O}}}_{2}+6{e}^{-}.$$

As a result, little aluminum was detected in the innermost TiO_2_ shell as shown at points “2” and “3” in Fig. 3(b). The generated Al^3+^ interstitial ions via Equ. (2) may escape via the outer mingled shell of TiO_2_ and Al_2_O_3_ to compensate the outermost aluminum-depleted shell in Stage 5, leading to its disappearance and a wider mingled shell (“Stage 6” in Fig. 6).

In conclusion, a minor addition of 0.1% yttrium in the TiAlNbCr alloy led to the formation of Y_2_O_3_ particles, together with B2-phase, γ-TiAl and α_2_-Ti_3_Al/γ-TiAl lamellar structures. The presence of Y_2_O_3_ particles was responsible for the occurrence of two distinctive characteristics of oxidation at high temperatures: core-multishell globular oxidation and “daisy” flower-like oxidation, which were observed for the first time, to the best of our knowledge. The globular oxide structures consisted of a core Y_2_O_3_ particle and multi-layered TiO_2_/Al_2_O_3_-rich/TiO_2_-rich shells from the inside to outside, while the flower-like inner oxide structures exhibited a core Y_2_O_3_ particle surrounded by the divergent Al_2_O_3_ and oxygen-rich α_2_-Ti_3_Al within the substrate in the neighborhood of oxide scale. As the scale-substrate interface moved inward, the inner flower-like oxide structures underwent increasingly more severe oxidation and eventually transformed into the globular oxide structures. We anticipate that the findings of unique oxidation characteristics and the understanding of relevant formation mechanisms pave the way for the exploration and development of advanced oxidation-resistant TiAl-based materials for the high-temperature applications.

## Materials and Methods

The titanium aluminide alloy selected in this study with a nominal chemical composition of Ti-44Al-4Nb-1.5Cr-0.5Mo-0.1B-0.1Y (in at.%, and hereafter referred to as TiAlNbCr alloy) was prepared by double vacuum consumable arc melting technique. The test specimens with a size of 10 × 10 ×3 mm^3^ were cut from the cast ingot by electro-discharge machining. The surfaces of the specimens were polished with 1200-grit SiC papers, and then cleaned ultrasonically in acetone for 15 min before isothermal oxidation in air at 900 °C. To avoid that one of the specimen surfaces came into contact with a flat crucible and ensure that all of the specimen surfaces were fully exposed to the air during isothermal oxidation tests, the specimens were first placed into the hollow ceramic cylinders positioned horizontally in a larger flat crucible. Then the crucible was transferred into the furnace. The specimens after oxidation were weighed for determining the weight gain, and subsequently mounted with resin and polished to a mirror-like surface. To increase electrical conductivity and image resolution, the polished specimens were carbon-coated before scanning electron microscope (SEM) and electron probe microanalysis (EPMA) examinations. Elemental distributions of titanium, aluminum, oxygen, nitrogen, niobium, chromium, molybdenum, boron and yttrium were detected through EPMA and the corresponding standard samples used for calibration were pure titanium, Al_2_O_3_, BN, pure niobium, pure chromium, pure molybdenum, pure boron and YP_4_O_12_, respectively. X-ray diffraction (XRD) was used to identify phases in the alloy with a diffraction angle (2*θ*) from 10° to 100° with a step size of 0.02° and 1 s in each step.
